# The Reciprocal Effect of Elevated CO_2_ and Drought on Wheat-Aphid Interaction System

**DOI:** 10.3389/fpls.2022.853220

**Published:** 2022-07-14

**Authors:** Haicui Xie, Fengyu Shi, Jingshi Li, Miaomiao Yu, Xuetao Yang, Yun Li, Jia Fan

**Affiliations:** ^1^Hebei Key Laboratory of Crop Stress Biology, College of Agronomy and Biotechnology, Hebei Normal University of Science and Technology, Qinhuangdao, China; ^2^The State Key Laboratory for Biology of Plant Diseases and Insect Pests, Institute of Plant Protection, Chinese Academy of Agricultural Sciences, Beijing, China

**Keywords:** *Triticum aestivum*, *Sitobion miscanthi*, elevated CO_2_, drought, nutritional quality, insect resistance

## Abstract

Due to the rising concentration of atmospheric CO_2_, climate change is predicted to intensify episodes of drought. However, our understanding of how combined environmental conditions, such as elevated CO_2_ and drought together, will influence crop-insect interactions is limited. In the present study, the direct effects of combined elevated CO_2_ and drought stress on wheat (*Triticum aestivum*) nutritional quality and insect resistance, and the indirect effects on the grain aphid (*Sitobion miscanthi*) performance were investigated. The results showed that, in wheat, elevated CO_2_ alleviated low water content caused by drought stress. Both elevated CO_2_ and drought promoted soluble sugar accumulation. However, opposite effects were found on amino acid content—it was decreased by elevated CO_2_ and increased by drought. Further, elevated CO_2_ down-regulated the jasmonic acid (JA) -dependent defense, but up-regulated the salicylic acid (SA)-dependent defense. Meanwhile, drought enhanced abscisic acid accumulation that promoted the JA-dependent defense. For aphids, their feeding always induced phytohormone resistance in wheat under either elevated CO_2_ or drought conditions. Similar aphid performance between the control and the combined two factors were observed. We concluded that the aphid damage suffered by wheat in the future under combined elevated CO_2_ and drier conditions tends to maintain the status quo. We further revealed the mechanism by which it happened from the aspects of wheat water content, nutrition, and resistance to aphids.

## Introduction

Elevated CO_2_ is identified as one major driving force of global climate change, and results in higher air temperature and altered precipitation patterns, which increase the area of arid areas (IPCC, 2014; Li et al., 2014). In the agroecosystem, the co-evolution of plants and insects is influenced by distinct environmental factors, such as CO_2_, soil moisture, and air temperature. Under complex climate change, to clarify the effects of multiple environmental factors—especially elevated CO_2_ alongside other factors on plant-insect interactions—is crucial for better understanding the damage as well as the occurrence trends of insect pests (Murray et al., 2013; Scherber et al., 2013; Zandalinas et al., 2018; Hervé et al., 2019; Wei et al., 2022).

Elevated CO_2_ changes the primary and secondary metabolism of plants by stimulating the photosynthetic rate (Zavala et al., 2017), which not only resulted in increased carbon:nitrogen ratio, but also changed the insect resistance in plants (Zavala et al., 2013; Sun et al., 2015, 2016; Guo et al., 2017; Johnson et al., 2022). Meanwhile, drought stress drives plants to develop a number of physiological responses as well, such as closing stoma, increasing carbohydrate and amino acid accumulation, and activating phytohormone signaling pathways (Mcdowell et al., 2008; Lee and Luan, 2012; Danquah et al., 2014; Ullah et al., 2018; Xie et al., 2021), which subsequently lead to the change in plant nutritional quality (water content and C/N ratio) as well as plant chemical defenses to insects (English-Loeb et al., 1997; Guo et al., 2016; Castagneyroi et al., 2018; Suarez-Vidal et al., 2019).

Combined effects of elevated CO_2_ and drought on different host plants and further plant-pest interaction vary (Roth et al., 1997; Nackley et al., 2018). This host plant-species-specific pattern is not surprising and is also found in other combined effects analyses such as combined warming and elevated CO_2_. Depending on the plant, warming treatment may weaken or strengthen the effect of elevated CO_2_ treatment (Johns and Hughes, 2002; Murray et al., 2013; Niziolek et al., 2013). However, elevated CO_2_ routinely promotes stomatal closureand root growth, and prolongs physiological activity, which is proven helpful in withstanding the negative impact of drought stress on dry biomass, water status, and water use efficiency (Wall, 2001; Roy et al., 2016; Li et al., 2017, 2019b; Miranda-Apodaca et al., 2018; Uddin et al., 2018).

Aphids are key economic pests for many crops that cause extensive feeding damage and transmit viruses (Blackman and Eastop, 2000; Züst and Agrawal, 2016). Both elevated CO_2_ and drought stress indirectly affect insects in two ways, namely, the changes in plant nutritional quality and its resistance to insects (Zavala et al., 2013; Xie et al., 2021). Both ways mediated by phytohormones are important factors affecting aphid feeding, growth, and population size (Sun et al., 2016). As sap-sucking insects, the soluble sugar and free nitrogen assimilation transferred in plant phloem are the main sources of carbon and nitrogen nutrients for aphids (Oehme et al., 2013). Recent studies have revealed that aphid feeding alters the content of phytohormones (e.g. abscisic acid, ABA; Jasmonic acid, JA; salicylic acid, SA), as well as the expression of defense genes (AOC, LOX, PAL, PR-1) related to JA and SA signaling pathways in plant tissues (Guo et al., 2012; Zhang et al., 2017). Change in phytohormone content is an important index for plant resistance to aphids, and the expression is generally induced by aphid feeding. These phytohormones' signaling pathways work together to regulate plant defense response to aphids. The resulting induced resistance is common in plants. However, the specific aphid resistance effect varies between species or even genotypes (Kempel et al., 2011; Züst and Agrawal, 2016).

In the growth and development of wheat, elevated CO_2_ and water stress are two constraints. This, in turn, can alter the metabolic rates, development, and aphids feeding performance. Elevated CO_2_ increased wheat starch, sucrose, glucose, total non-structure carbohydrates (TNCs), TNC:nitrogen ratio, free amino acids, soluble protein and grain weight, and decreased fructose and nitrogen contents, which further enhanced the grain aphid *Sitobion avenae* Fabricius population fed on wheat (Chen and Ge, 2004), but decreased fecundity of bird cherry-oat aphid *Rhopalosiphum padi* (Navarro et al., 2020). Drought stress significantly promoted amino acid accumulation, soluble sugar, and phytohormone contents in wheat, and also increased aphid abundance and population growth rates fed on wheat (Mousa et al., 2019; Cui et al., 2020; Xie et al., 2021). Other research has shown that compared to semiarid and moist area clones, arid area clones of the aphid *Sitobion avenae* (Fabricius) tended to have longer developmental times and smaller body sizes (Ahammed et al., 2016). However, what the interaction between these two factors is and how it affects the susceptibility of wheat plants to insect attack is still completely occluded.

In the present study, we used a plant-aphid interaction system, namely wheat (*Triticum aestivum*) and its key pest aphid *Sitobion miscanthi*, which is widely misreported as *S. avenae* in China (Zhang, 1999; Jiang et al., 2019), to comprehensively and systematically investigate the combined effects of elevated CO_2_ and drought stress on wheat nutritional quality and resistance to aphid, as well as how these changes in the host plants affect aphid performance. Results from this study should help to understand the occurrence trend of aphid population on wheat under elevated CO_2_ and drought, which will subsequently aid in adjusting pest control strategies under the context of future climate change.

## Materials and Methods

### Aphids

The grain aphid isolate *S. miscanthi* was originally collected from wheat in the Hebei province of China. This isogenic colony was started from a single parthenogenetic female and was maintained on wheat (*Triticum aestivum*) in the laboratory at 22 ± 1°C, with 75% relative humidity and 16L: 8D photoperiod. To avoid overcrowding, aphids were continuously transferred to new plants until the start of the experiment.

### Plant Preparation and Treatment With Elevated CO_2_ and Drought

Elevated CO_2_ conditions were supplied from gas tanks. Ambient CO_2_ conditions were supplied from the surrounding air entering the environmental chamber facilities. The chambers were maintained at 25 ± 1°C, 60–70% relative humidity, and 14 h light /10 h dark photoperiods with 9000 lx fluorescent lamp active radiation levels. Wheat seeds (variety Zhongmai 175) were planted in pots (7.5 cm diameter x 9.0 cm height) with a sterilized loamy field soil (organic carbon content ~75 g kg^−1^). Each pot was planted with several seeds, and then only one seedling kept for the coming treatments.

Closed-dynamic CO_2_ chambers (CDCC) were used and the parameters set here mainly followed several classic cases which used CDCC (Chen and Ge, 2004; Guo et al., 2012; Kurepin et al., 2018; Dusenge et al., 2020). Six chambers were employed, and three of them were treated with elevated CO_2_ (~750 ± 15 ppm), the other three as their controls were maintained at ambient CO_2_ (~380 ppm). Wheat plants were exposed to the CO_2_ and drought treatments from germination to the end of the experiment. Forty pots were used for well-watered (80% of field capacity moisture) and drought (40% of field capacity moisture) treatment, respectively, in each chamber. Wheat plants (2-week-old) from half of 40 pots were collected for testing physiological indicators (one leaf each pot) after treatment, and the remaining 20 pots were further treated with aphid infestation. The testing indicators were performed in three replicates. To minimize positional effects, plants were randomly positioned within each chamber daily.

### Relative Water Content Measurement

Leaf relative water content (RWC) was measured using the formula RWC (%) = (fresh weight-dry weight)^*^100/ (turgor weight-dry weight) (Barrs and Weatherley, 1962). Fresh weight (FW) was measured and leaves were cut off to rehydrate in distilled water for 24 h at 15°C in darkness to obtain the weight at full turgor (TW). Leaf dry weight (DW) was obtained after the samples were dried in an oven at 70°C for 48 h.

### Soluble Sugar, Amino Acid, and Phytohormones Measurement

To quantify soluble sugar and amino acid concentrations in the phloem, phloem exudate from leaves was obtained using the EDTA exudation technique previously described by Tetyuk et al. (2013). For soluble sugar analysis, phloem exudate from leaves and 40% acetonitrile (40 mL) were transferred into volumetric flasks (50 ml), followed by ultrasound extraction for 30 min and dilution with 40% acetonitrile to 50 ml for use as samples. Samples of 20 μL aliquots from each treatment were injected into the HPLC-MS/MS system. Sugars were separated with a Waters BEH Amide column (4.6 mm × 250 mm, 5 μm; Waters Corporation) using 75% acetonitrile as the mobile phase with isocratic elution at a flow rate of 1.0 ml/min. The column was maintained at 30°C. For amino acid analysis, phloem exudates and pure water (60 ml) were transferred into 100 mL volumetric flasks. After ultrasonic extraction for 30 min, the volume was fixed to the scale mark with water. Then, 10 μL aliquots of each sample were injected into the HPLC-MS/MS system. Amino acids were separated using an ACQUITY HSS T3 column (100 mm × 2.1 mm, 1.8 μm; Waters Corporation) under gradient conditions with 5 mM ammonium acetate (A) and acetonitrile (B) as the mobile phases at a 0.3 ml/min flow rate. The gradient program is shown in Supplementary Table S1. The column was maintained at 30°C.

For the phytohormone (ABA, JA, and SA) defense response analysis, the above wheat leaves with and without aphid infection were collected. Twenty-two wingless adults were transferred to wheat leaves for 24 h at the two-leaf stage. Then, all aphids were removed. Leaves without aphid infection were used as control. Samples of leaves (0.2 g) were homogenized by liquid nitrogen. The resulting homogenate and 10 mL of ethyl acetate were transferred into a centrifuge tube, followed by ultrasound extraction for 20 min and centrifugation for 10 min at 20,000 × g. The supernatant was evaporated to dryness under a stream of nitrogen at 40°C, and the final extracts were dissolved in 1 mL of 70% methanol and used as samples for analysis. Then, 10 μL aliquots of the samples were injected into the HPLC-MS/MS system. Phytohormones were separated with an Acquity UPLC® BEH C18 column (2.1 mm. 100 mm 1.7 μm; Waters Corporation) under gradient conditions, using 0.1% formic acid (A) and methanol (B) as the mobile phases, at a flow rate of 0.3 ml min^¯1^. The gradient program for phytohormone quantification is shown in Supplementary Table S2. The column was maintained at 30°C.

### Gene Expression Detection by RT-qPCR

Total RNA was extracted using Trizol reagent (Invitrogen, Carlsbad, CA, USA) and combined with a micro total RNA extraction kit (Tianmo, Beijing, China) following the manufacturer's instructions. The tissues were homogenized with a liquid-nitrogen-cooled mortar and ground with a pestle into a very fine dust. Homogenized tissues were covered with 1 mL of Trizol reagent. RNA degradation and contamination were monitored on 2% agarose gels. RNA purity was checked using a Nanodrop ND-1000 spectrophotometer (NanoDrop products, Wilmington, DE, USA). RNA concentration was measured using a spectrophotometer RNA Nano 6000 Assay Kit of the Agilent Bioanalyzer 2100 system (Agilent Technologies, CA, USA). Individual total RNA was isolated and corresponding type cDNA was synthesized using the TRUEscript RT kit (LanY Science & Technology, Beijing, China) following the manufacturing protocol.

When plants were at the two-leaf stage, 20 wingless *S. miscanthi* adults were transferred to the first leaf (i.e., the oldest leaf) of each plant (Zhang et al., 2017). Plants without aphid feeding were set as control. After feeding for 24 h, all aphids were removed. The relative expressions of four genes in leaves, *Lipoxygenase* (*LOX*) and *allene oxide synthase* (*AOS*) for the JA-responsive pathway (Liu et al., 2011) and the SA synthesis enzyme gene *phenylalanine ammonia lyase* (*PAL*) and the induced SA marker protein gene *pathogenesis-related protein 1* (*PR-1*) for the SA-responsive pathway (Chen et al., 2009) were determined using RT-qPCR. Actin was used as the internal control and was amplified using the primer sequences described by Liu et al. (2011). Referring to Zhang's primers (Supplementary Table S3, 2017), RT-qPCR was performed on an ABI 7500 Real-Time PCR System (Applied Biosystems, Foster City, CA, USA). The PCRs were performed in 20-μL reaction volumes that contained 1 μL of cDNA, 0.5 μL each of 10 μmol L^−1^ forward and reverse primers, 10 μL of 2× SybrGreen qPCR Mastermix, and 8.0 μL of ddH_2_O under the following thermal cycling conditions: 2 min at 95°C followed by 40 cycles of 10 s at 95°C and 30 s at 60°C.

### Aphid Life Table Parameter Measurement

Wheat plants under the different treatments mentioned above were used to rear aphids in corresponding environmental chambers. Using a camel hair brush, a single newly emerged nymph (<6 h) was placed on the first leaf of each plant (20 aphids in total for each treatment). To prevent the aphids from escaping, the plant was confined in transparent plastic column cages covered with double-deck gauze on top. All indicators were tested in triplicate. To minimize positional effects, plants were randomly positioned within each chamber daily. The above-recorded data was used to construct a life table and obtain the life table parameters (net reproductive rate *R*_0_, intrinsic rate of increase *r*_m_, generation time *T*, and finite rate of increase λ). Age-specific reproduction was used to construct a life table (Birch, 1948).

### Statistical Analysis

For RT-qPCR, the fold changes in the expression of target genes were computed using the 2^−ΔΔCt^ normalization method (Livak and Schmittgen, 2001). For the life table parameters, *r*_m_ was computed using the Euler equation:$\overset{\infty }{\underset{x=0}{\sum }}e^{rx}l_{x}m_{x}=1$, where *l*_*x*_ is survivorship of the original cohort over the age interval from day *x*-1 to day *x* (i.e., pivotal age) and *m*_*x*_ is the mean number of offspring produced per surviving aphid during the age interval *x* (Maia et al., 2000). *R*_0_, *T*, and λ, were calculated as reported by Maia et al. (2000). The effects of elevated CO_2_ and drought stress on relative water content, soluble sugar content, and free amino acids content as well as aphid life table parameters were tested by two-way ANOVAs (SAS Institute, Cary, NC, USA). The effects of elevated CO_2_, drought, and aphid infestation on phytohormone (ABA, JA, and SA) content as well as JA- and SA-related gene expression in wheat were tested by three-way ANOVAs. Least significant different (LSD) tests were used to determine if treatment means significantly differed when ANOVAs indicated a factor was significant. For all analyses, *P* < 0.05 was considered the threshold for statistical significance.

## Results

### Water Content in Wheat

**Figure 1:**
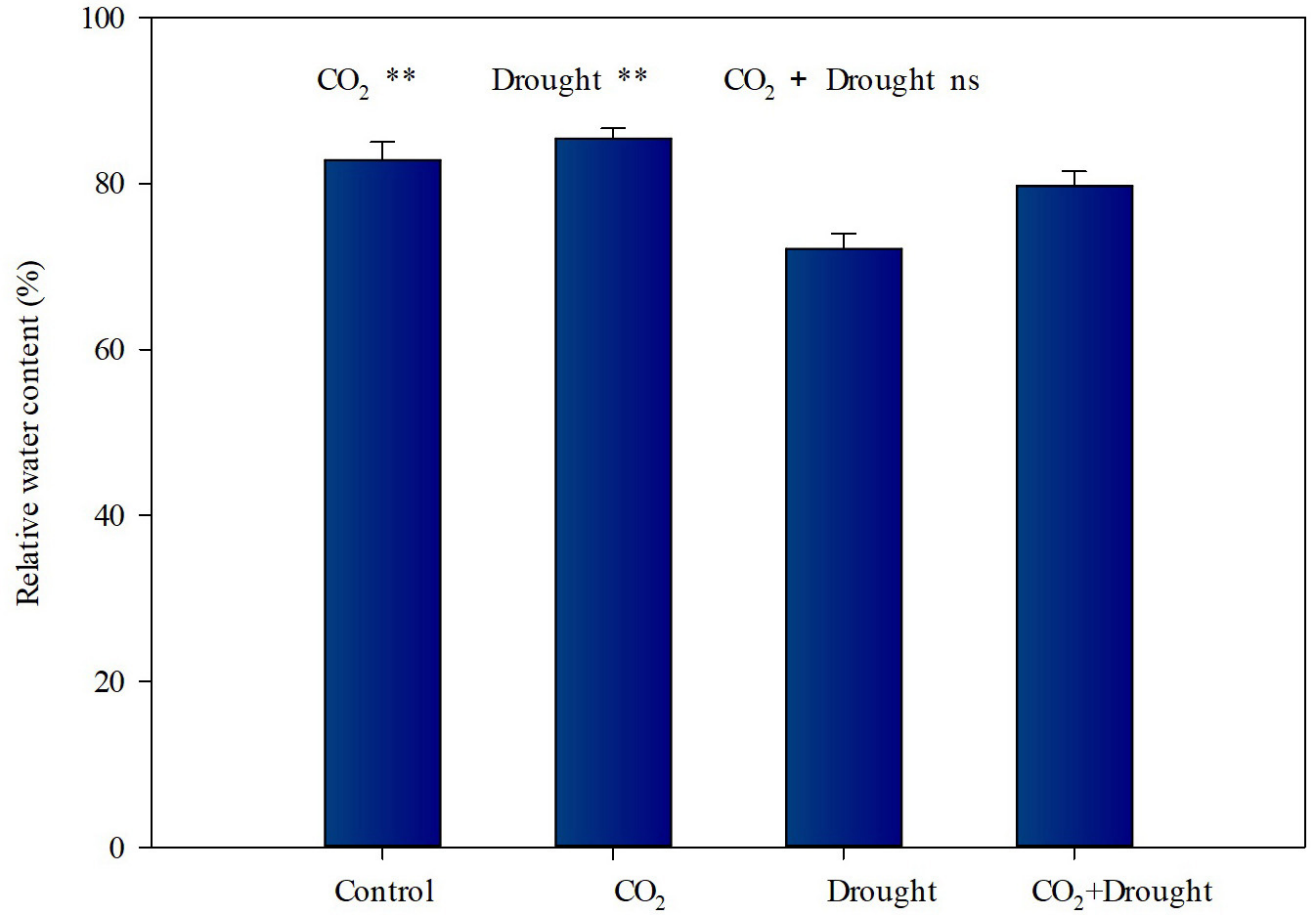
Relative water content of *Triticum aestivum* grown under elevated CO_2_ and drought conditions. Each value shown represents the mean (±SE) of three replicates. *P* values are provided for two-way ANOVA on the effects of elevated CO_2_ and drought treatments on relative water content. Significant differences: *P < 0.05; **P < 0.01; ns, *P* > 0.05.

The relative water contents of wheat under elevated CO_2_ or drought stress vary in opposite ways (Figure 1, Supplementary Table S4). It increased by 6.63% under elevated CO_2_ conditions (Figure 1), and decreased by 10.9% under drought stress conditions (Figure 1). The interactions between elevated CO_2_ and drought stress on the relative water content of wheat were not significant (Supplementary Table S4).

### Soluble Sugar Content in Wheat

**Figure 2:**
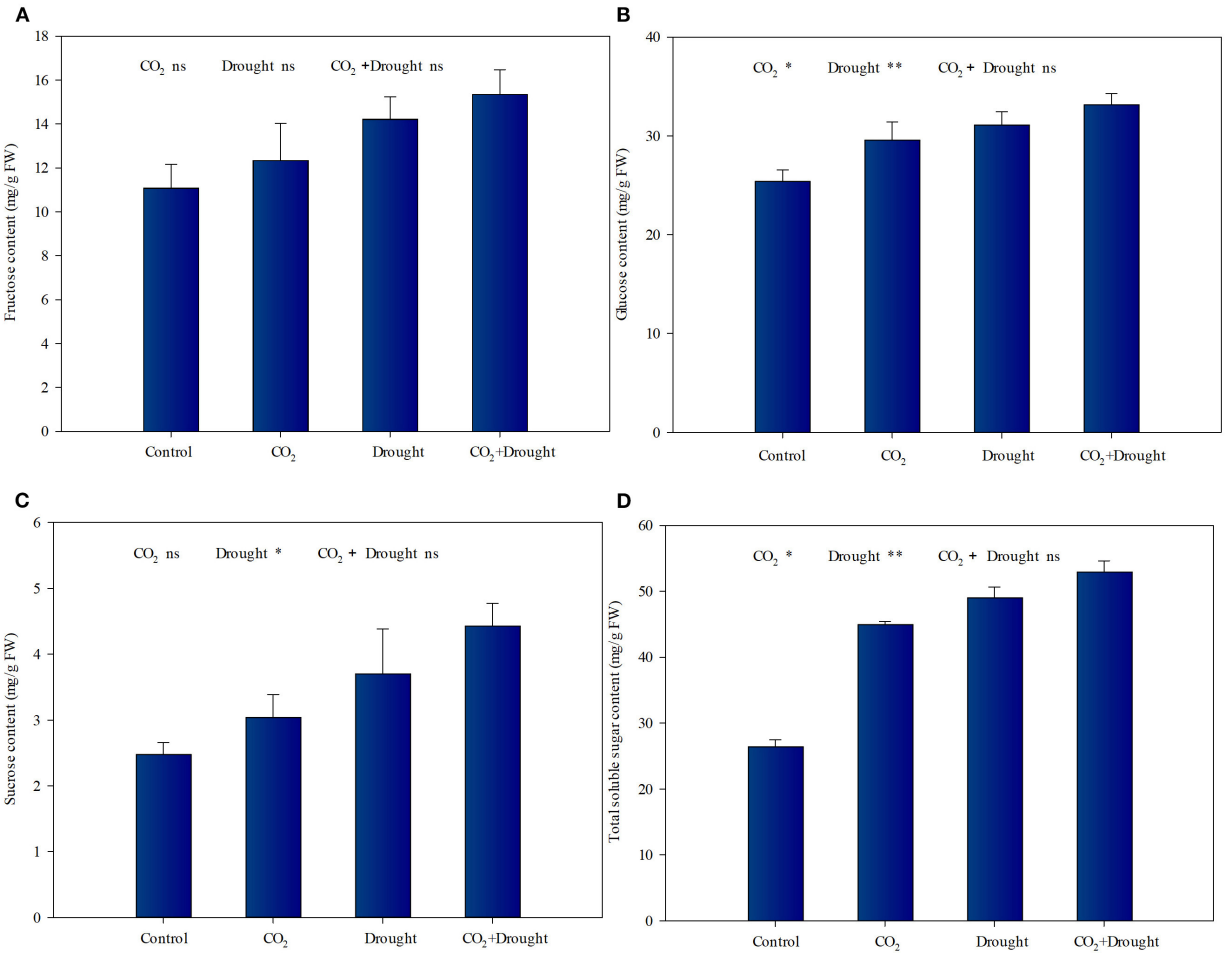
Soluble sugars content of *Triticum aestivum* grown under elevated CO_2_ and drought conditions. **(A)** fructose; **(B)** glucose; **(C)** sucrose; **(D)** total soluble sugars. Each value shown represents the mean (±SE) of three replicates. *P-*values are provided for two-way ANOVA on the effects of elevated CO_2_ and drought treatments on soluble sugar content. Significant differences: *P < 0.05; **P < 0.01; ns, *P* > 0.05.

Elevated CO_2_ and drought stress significantly promoted soluble sugar accumulation in wheat (Figure 2, Supplementary Table S5). Relative to the ambient CO_2_ treatment, both the glucose and total soluble sugar content increased under elevated CO_2_ conditions (Figures 2B,D). Meanwhile, Relative to the well-watered treatment, the fructose, glucose, sucrose, and total soluble sugar content increased in wheat under drought stress (Figures 2A–D). The interaction effects between elevated CO_2_ and drought stress on soluble sugars in wheat were not significant (Supplementary Table S5).

### Amino Acid Content in Wheat

**Figure 3:**
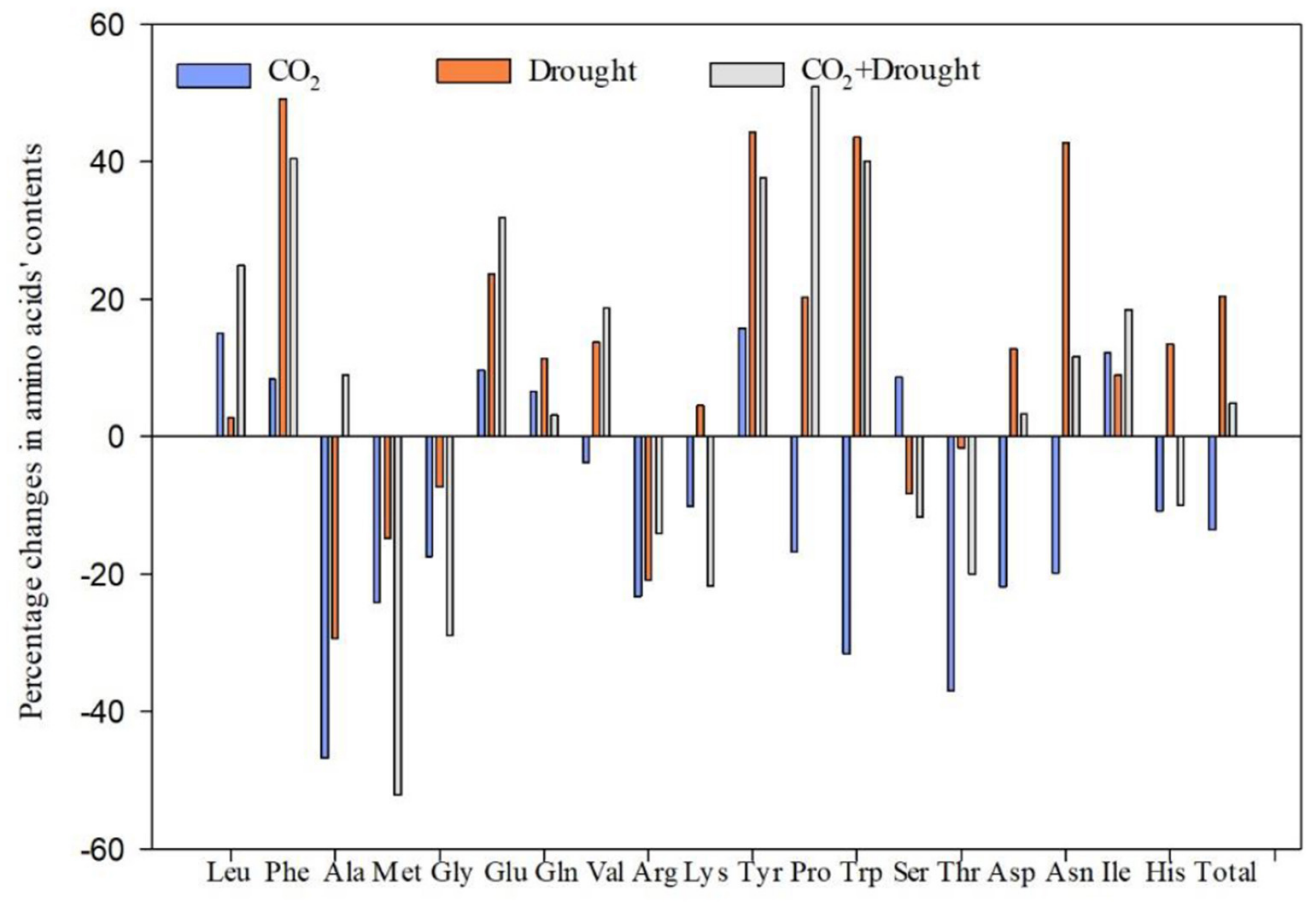
Percentage changes in amino acid content of *Triticum aestivum* grown under elevated CO_2_ and drought conditions. Percentage change value (%) = (treatment-control)*100/control.

Nineteen amino acids in wheat were quantified (Figure 3, Supplementary Table S6). The effects of elevated CO_2_ and drought stress on amino acid accumulation in wheat were different and some indicators even show opposite trends. Elevated CO_2_ significantly decreased the Met, Gly, Lys, Try, Thr, Asp, His, and total amino acid content compared to ambient CO_2_ treatment (Supplementary Table S6). Drought stress significantly increased the Phe, Glu, Tyr, Pro, Try, Asp, Asn, and total amino acid content, compared to the well-watered treatment (Supplementary Table S6). Furthermore, it showed significantly negative interaction effects between elevated CO_2_ and drought stress on three amino acids including alanine and tryptophan (Supplementary Table S6).

### Phytohormone-Dependent Defense Against Aphids

**Figure 4:**
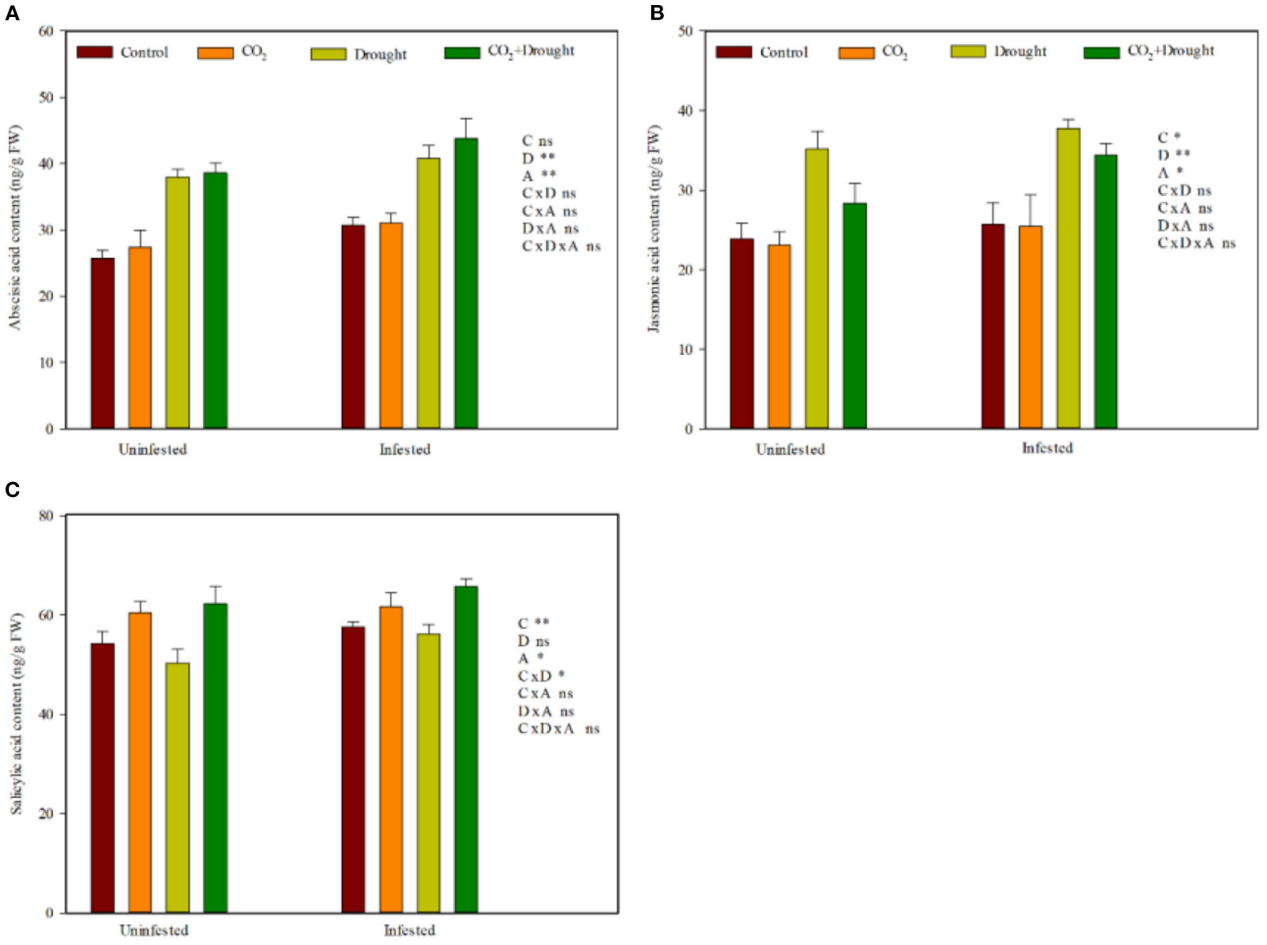
Phytohormone content of *Triticum aestivum* grown under elevated CO_2_ and drought conditions with and without aphid infestation. **(A)** abscisic acid; **(B)** jasmonic acid; **(C)** salicylic acid. Each value shown represents the mean (±SE) of three replicates. *P-*values are provided for three-way ANOVA on the effects of elevated CO_2_ **(C)**, drought **(D)**, and aphid **(A)** treatments on phytohormone content. Significant differences: *P < 0.05; **P < 0.01; ns, *P* > 0.05.

Elevated CO_2_, drought stress, and aphid infestation significantly influenced the wheat phytohormone content (Figure 4, Supplementary Table S7). Relative to the ambient CO_2_ treatment, elevated CO_2_ decreased JA, but increased SA content (Figures 4B,C). Relative to the well-watered treatment, drought stress increased ABA and JA content (Figures 4A,B). Aphid infestation significantly increased ABA, JA, and SA content in wheat compared with control (the uninfested wheat) (Figures 4A–C). Moreover, the interaction effects among elevated CO_2_, drought stress, and aphids infestation on wheat phytohormones were all not significant (Supplementary Table S7).

**Figure 5:**
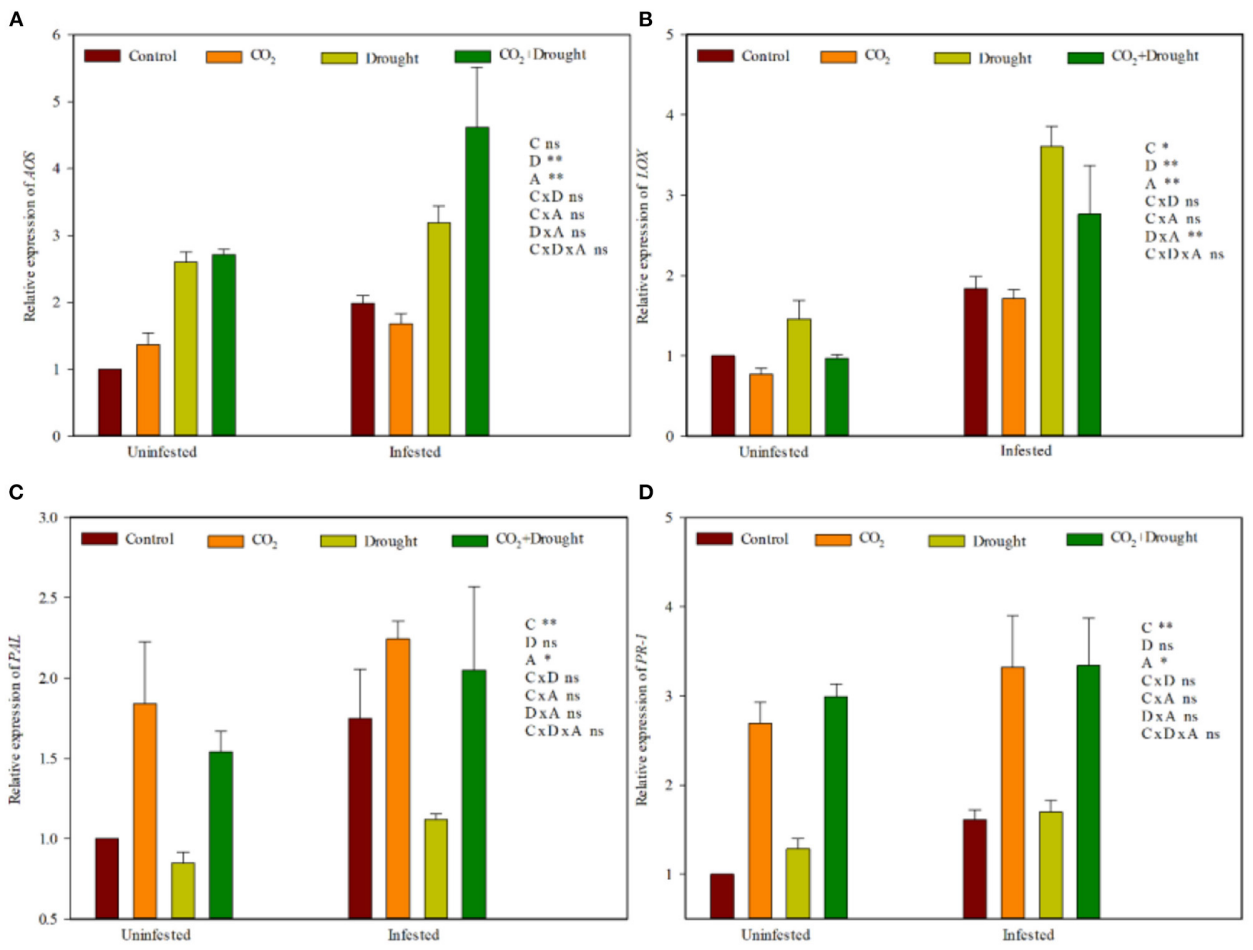
JA- and SA-related gene expression of *Triticum aestivum* grown under elevated CO_2_ and drought conditions with and without aphid infestation. **(A)** *allene oxide synthase* (*AOS*); **(B)** *Lipoxygenase* (*LOX*); **(C)** *phenylalanine ammonia lyase* (*PAL*), **(D)** *pathogenesis-related protein 1* (*PR-1*). Each value shown represents the mean (±SE) of three replicates. *P-*values are provided for three-way ANOVA on the effects of elevated CO_2_ **(C)**, drought **(D)**, and aphid **(A)** treatments on JA- and SA-related gene expression. Significant differences: *P < 0.05; **P < 0.01; ns, *P* > 0.05.

Furthermore, two genes from each plant hormone defense response pathway (JA and SA) in response to aphid induction in wheat were quantified by RT-qPCR. Elevated CO_2_, drought stress and aphid infestation significantly influenced the relative expression of four defense-related genes (Figure 5, Supplementary Table S8). *LOX* (JA) was down-regulated, and *PR-1*, as well as PAL (SA), were up-regulated in wheat grown under elevated CO_2_ (Figures 5B–D). Relative to the well-watered treatment, the relative expressions of *AOS* (JA) and *LOX* (JA) were up-regulated in wheat grown under drought stress (Figures 5A,B). Aphid infestation up-regulated the relative expression of *AOS, LOX* (JA), *PAL*, and *PR-1* (SA) in wheat, compared to control (the uninfested wheat) (Figures 5A–D). Three factors as well as any two among the three factors analyzed on four gene expressions showed no interaction effect except drought stress and aphid infestation on *LOX* which showed a significantly positive interaction effect (Supplementary Table S8).

### Changes in Performance of Aphid Populations on Wheat

**Figure 6:**
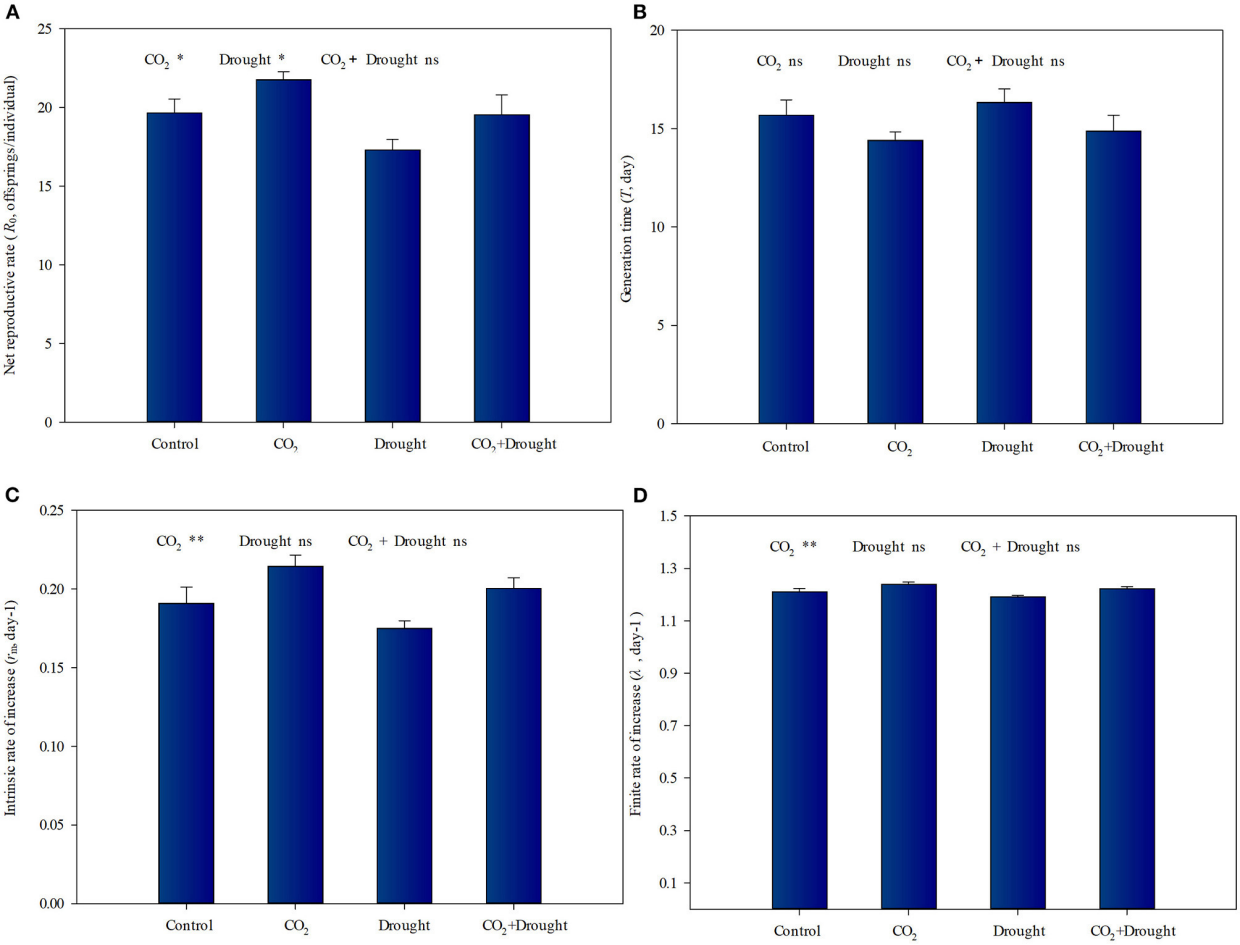
Life table parameters of aphid reared on *Triticum aestivum* grown under elevated CO_2_ and drought conditions. **(A)** net reproduction rate (*R*_0_); **(B)** generation time (*T*); **(C)** intrinsic rate of increase (*r*_m_); **(D)** finite rate of increase (λ). Each value shown represents the mean (±SE) of three replicates. *P-*values are provided for two-way ANOVA on the effects of elevated CO_2_ and drought treatments on life table parameters. Significant differences: *P < 0.05; **P < 0.01; ns, *P* > 0.05.

There are contrasting effects of elevated CO_2_ and drought stress on the life table parameters of aphids fed on wheat (Figure 6, Supplementary Table S9). Under elevated CO_2_, the *R*_0_, *r*, and λ values of aphid populations increased by 10.6, 13.1, and 2.5%, respectively, compared to control (ambient CO_2_) (Figures 6A,C,D). The *R*_0_ values of aphid populations decreased by 12.4% under drought stress compared to the well-watered treatment (Figure 6A). The interaction effects between elevated CO_2_ and drought stress on the aphid life table parameters were not significant (Supplementary Table S9).

## Discussion

The water content, nutritional quality (soluble sugar, amino acid, etc), and the secondary metabolic defense pathways in host plants are key limiting factors for aphids' growth and development (Douglas, 1993; Bezemer and Jones, 1998; Mewis et al., 2012; Züst and Agrawal, 2016). The effects of many single environmental factors on all the above aspects have been well-documented. It is necessary and interesting to further explore how the combined environmental factors such as elevated CO_2_ and drought stress work on the aphids' limiting factors as well as further change the plant-aphid interactions.

The Combined Effects of Elevated CO_2_ and Drought Treatment on Relative Water Content Tend to Balance Out.

Elevated CO_2_ has been found to induce stomatal closure, which leads to lower evaporative flux density. As a result of decreases in evaporative flux density and increases in net photosynthesis, also found to occur in elevated CO_2_, plants have often been shown to maintain higher water use efficiencies when grown under elevated CO_2_ conditions (Barbehenn et al., 2004; Sun et al., 2015). The elevated CO_2_ also can facilitate the access to sub-soil water by promoting wheat root growth (Li et al., 2017; Uddin et al., 2018). In the present study, we also found that elevated CO_2_ treatment increased the water content of wheat. Meanwhile, drought stress decreased the water content as expected. Further, there was no significant interaction between these two environmental factors which indicates that their combined effects can be regarded as the simple sum of their single effects. Thus, the similar water contents between control and combined elevated CO_2_ and drought treatment demonstrated that elevated CO_2_ alleviates lower water content caused by drought stress.

### The Combined Effects Promote Carbohydrate Accumulation

It has long been recognized that elevated CO_2_ increases the photosynthetic rate of C_3_ plants including wheat, and further promotes carbohydrate accumulation (Barbehenn et al., 2004; Chen et al., 2005). La et al. (2019) also indicated that plant sugar accumulation was mainly due to the increased sucrose content with the highest expression of ABA-dependent sucrose signaling genes under drought stress. To be specific, in wheat the enhancements in carbohydrate accumulation of each single factor such as elevated CO_2_ (Sun et al., 2009) and drought (Xie et al., 2021) have been verified. Also, under certain combined abiotic stresses such as drought and warming up, the increased carbohydrate accumulation had been reported (Zandalinas et al., 2018). Drought stress not only increased the energy accumulation but also coordinated with the activation of specific physiological and molecular responses in growing plants to mitigate the damaging effects of drought (Zandalinas et al., 2018). In the present study, our results supported that each factor of elevated CO_2_ and drought stress significantly promoted soluble sugar accumulation in wheat.

### The Combined Effect on Nitrogen Concentration Tends to Balance Out

Lower nitrogen concentration was generally found in the plants grown under elevated CO_2_ (Zavala et al., 2013). Indeed, in the present study, all tested contents such as Met, Gly, Lys, Try, Thr, Asp, His, and total amino acid content of wheat were decreased under elevated CO_2_. There have been several explanations for this decline phenomenon. It may be due to enhanced carbohydrates or biomass accumulation in plants grown under elevated CO_2_ conditions, then the amino acid concentration was diluted (Nie et al., 1995; Smart et al., 2010; Novriyanti et al., 2012). Besides, the elevated CO_2_ inhibits plant nitrogen assimilation and metabolism (Bloom et al., 2014) and further mechanism study revealed that the reduced activity of nitrate reductase finally coursed the decreased protein and amino acid contents (Dier et al., 2017; Andrews et al., 2019).

On the contrary, drought stress increased the amino acid content in this study. Previous studies have shown that the accumulation of amino acids enhances plant drought stress tolerance by regulating the activation of material metabolism in plants (Suguiyama et al., 2014). Interestingly, three up-regulated amino acids under drought stress in the present study, namely Try, Tyr, and Phe, are located in the downstream of the shikimic acid pathway, which precisely functions to adjust metabolism toward secondary metabolite production and contributes to hormone metabolism in wheat (Tzin and Galili, 2010).

Their opposing effects on total amino acid contents as well as most of the other test amino acids (17 of 19) in wheat of elevated CO_2_ and drought did not show interaction, except for two amino acids, Ala and Try. The interaction indicated the existence of a potential crosstalk in which they both participate and a more complicated mechanism of change patterns in certain amino acids and proteins under combined elevated CO_2_ and drought treatment.

### Various Phytohormone-Dependent Wheat Defenses Against Aphids

Plant response to abiotic and biotic stress commonly through ABA, JA, or SA defense signaling pathways including the accumulation of phytohormone and up-regulated related gene expression (Adie et al., 2007; Danquah et al., 2014; Zandalinas et al., 2016; Gupta et al., 2017), although the regulated process is complicated and difficult to parse. In general, the ABA signaling pathway activity up-regulated the JA signaling pathway but suppressed the SA signaling pathway when plants were grown under abiotic stress. The up-regulated JA further enhanced the effective resistance of host plants to herbivorous insects (Casteel et al., 2008; Zavala et al., 2008; Ahammed et al., 2016; Guo et al., 2016). JA and SA defense-related gene expression (*LOX, AOS, PR-1*, and *PAL*) studies showed that infiltration of *S. avenae* watery saliva in wheat leaves induced the JA and SA-dependent defense (Zhang et al., 2017).

Elevated CO_2_ weakens the effective resistance of wheat to aphids by altering the phytohormone signal pathways (Guo et al., 2012, 2016; Sun et al., 2013, 2015). In the present study, elevated CO_2_ down-regulated the expression levels of the JA defense-related gene *LOX* and further reduced JA accumulation. Meanwhile, it up-regulated the expression levels of the SA defense-related genes *PR-1* and *PAL* and further increased SA accumulation, which indicated that elevated CO_2_ weakeed the resistance of wheat.

Drought stress enhanced ABA accumulation and promoted the activity of key enzymes from JA signaling pathway and therefore increased the resistance against aphids in wheat. In the present study, drought stresses up-regulated the expression of *LOX* and *AOS* from the JA pathway in wheat and increased ABA and JA accumulation, which showed that drought stress strengthens the resistance of wheat.

Moreover, aphid infestation always up-regulated the JA and SA signaling pathways by increasing JA and SA accumulation and their defense-related genes (*LOX, AOS, PR-1*, and *PAL*) regardless of the elevated CO_2_ and drought conditions in the present study (Supplementary Tables S6, 7).

In summary, the wheat resistance to aphids produced by phytohormone under elevated CO_2_, drought stresses, or aphid infestation is quite different. But the combined effects among all three factors tended to beindependent, and there was no interaction at all.

### Changes in Wheat—Aphid Interaction Under Elevated CO_2_ and Drought Stresses

Higher host plant water availability enhances aphid feeding efficiency (Sun et al., 2015; Guo et al., 2016). However, aphid response varied to the increasing soluble sugar in their host plants. Some reports have suggested that the increasing soluble sugar content in host plants is beneficial to aphid feeding (Li et al., 2019a). However, others showed that increased soluble sugar content is not necessarily conducive to aphid growth (Slosser et al., 2004; Alkhedir et al., 2013).

Aphids utilize a food source rich in carbohydrates but relatively low in nitrogen. Even so, they must obtain some essential amino acids such as Leu, His, Lys, etc from their host (Chen et al., 2005). Thus, the nitrogen concentration of the host is commonly a limiting nutritional factor for aphids, as reflected in the strong correlations between this variable (nitrogen concentration of wheat) and the individual performance of aphids (Sandström and Pettersson, 1994; Hansen and Moran, 2011). Other literature further shows that the decline of amino acid content enhanced aphid ingestion efficiency which was driven by their instinct for nitrogen (Chen et al., 2005; Sun et al., 2009). Our work here supported the above ideas. Considering the increased soluble sugar accumulation and decreased total amino acid content of wheat under elevated CO_2_, the improved indicators such as *R*_0_, *r*, and λ values of aphid populations could benefit from the resulting increased feeding effectiveness. Besides, the increased water content also enhanced aphid feeding efficiency, which together with weakened JA-dependent defense for aphids explained why elevated CO_2_ improved the occurrence of aphid populations in wheat.

However, the *R*_0_ value of aphid populations decreased when they fed on wheat grown under drought stress. The decreased fecundity may be relative to the lower water content reducing aphid feeding efficiency and higher JA-dependent defense in wheat grown under drought stress, although drought increased the soluble sugar and amino acid accumulation in wheat. At the same time, the existing studies also showed that the increased JA and SA content enhanced the accumulation of sugars and amino acids in plants (Ilyas et al., 2017; Zhao et al., 2021). Thus, both the accumulation of phytohormone and substances may contribute to the stress resistance (drought and aphid) of wheat in this study, which could be explained through further experiment.

Although each of the two factors significantly influenced wheat water contents, nutritional quality, and phytohormone-dependent defense, further indirectly influencing the performance of aphids significantly, the combined effect on the aphid life table parameters was not significant. It showed similar aphid performance between control and combined treatment in this study, which suggested that in the case of elevated CO_2_ and drought stresses, aphid damage will continue.

We concluded that the aphid damage suffered by wheat in the future under combined elevated CO_2_ and drier conditions tends to maintain the status quo. We further revealed the mechanism by which it happened from aspects of wheat water content, nutrition, and resistance to aphids. It could be necessary to reveal details of the plant-insect interactions from the perspective of multiple environmental factors and species specificity and deepen our understanding of the relationship between pest occurrence and its damage to plants.

## Data Availability Statement

The original contributions presented in the study are included in the article/Supplementary Material, further inquiries can be directed to the corresponding author/s.

## Author Contributions

HX and JF conceived the idea and developed research questions. FS, YL, and HX collected and analyzed the data and prepared the original draft. MY, JL, and XY managed the experiment, collected the data, and reviewed and edited the manuscript. All authors contributed to the article and approved the submitted version.

## Funding

This work was supported by the Science and Technology Research Project for Colleges and Universities in Hebei Province (BJ2020049) and the National Natural Science Foundation of China (31871966).

## Conflict of Interest

The authors declare that the research was conducted in the absence of any commercial or financial relationships that could be construed as a potential conflict of interest.

## Publisher's Note

All claims expressed in this article are solely those of the authors and do not necessarily represent those of their affiliated organizations, or those of the publisher, the editors and the reviewers. Any product that may be evaluated in this article, or claim that may be made by its manufacturer, is not guaranteed or endorsed by the publisher.
