# Supplementary material for: The Reciprocal Effect of Elevated CO2 and Drought on Wheat-Aphid Interaction System
Source: Front Plant Sci. 2022 Jul 14;13:853220. doi: 10.3389/fpls.2022.853220 (PMC9330134; doi:10.3389/fpls.2022.853220)
Supplement: Supplementary file 1 [file Data_Sheet_1.docx]

**Table S1.** Gradient elution program in the HPLC analysis for amino acids.

| Time / min | A / % | B / % |
| --- | --- | --- |
| 0 | 100 | 0 |
| 3.0 | 100 | 0 |
| 3.50 | 95 | 5 |
| 4.50 | 95 | 5 |
| 5.00 | 70 | 30 |
| 6.00 | 70 | 30 |
| 6.50 | 30 | 70 |
| 7.5 | 30 | 70 |
| 8.00 | 100 | 0 |
| 12.0 | 100 | 0 |

**Table S2** Gradient elution program in the HPLC analysis for phytohormone.

| Time / min | A / % | B / % |
| --- | --- | --- |
| 1 | 75 | 25 |
| 1.5 | 35 | 65 |
| 3 | 35 | 65 |
| 3.5 | 75 | 25 |
| 6 | - | - |

**Table S3.** RT-qPCR primers for genes involved in JA and SA defense responses.

| Primer | Primer sequences | E value |
| --- | --- | --- |
| *AOS*-F | ACTTCAACACGCTCAACGACT | 0.92 |
| *AOS*-R | TCACCGCTGACAAAGATGG |  |
| *LOX*-F | GACCAGCGAAACAACAACC | 0.96 |
| *LOX*-R | GCATACAATAGCGGGAACAC |  |
| *PAL*-F | CCACCCTGGACAGATTGAA | 0.98 |
| *PAL*-R | ATGAGCGGGTTGTCGTTG |  |
| *PR-1*-F | ATAACCTCGGCGTCTTCAT | 1.01 |
| *PR-1*-R | TACTCGCTCGGTCCCTCT |  |
| *Actin*-F | GGAAAATCAGTCTCGGTTCAG | 0.96 |
| *Actin*-R | TCATACAGCAGGCAAGCAC |  |

**Table S4.** Summary of ANOVA results for effects of elevated CO_2_ and drought on relative water content.

| Treatment | *df* | *F* | *P* |
| --- | --- | --- | --- |
| CO_2_ | 1 | 10.13 | 0.01 |
| Drought | 1 | 26.12 | < 0.01 |
| CO_2_*drought | 1 | 2.48 | 0.15 |

**Table S5.** Summary of ANOVA results for effects of elevated CO_2_ and drought on soluble sugar contents.

| Measurement | Treatment | *df* | *F* | *P* |
| --- | --- | --- | --- | --- |
|  | CO_2_ | 1 | 0.93 | 0.36 |
| Fructose | Drought | 1 | 6.12 | 0.04 |
|  | CO_2_*drought | 1 | 0.01 | 0.95 |
|  | CO_2_ | 1 | 5.93 | 0.05 |
| Glucose | Drought | 1 | 11.76 | <0.01 |
|  | CO_2_*drought | 1 | 0.61 | 0.46 |
|  | CO_2_ | 1 | 2.25 | 0.17 |
| Sucrose | Drought | 1 | 9.27 | 0.02 |
|  | CO_2_*drought | 1 | 0.04 | 0.85 |
|  | CO_2_ | 1 | 5.31 | 0.05 |
| Total sugars | Drought | 1 | 22.73 | <0.01 |
|  | CO_2_*drought | 1 | 0.01 | 0.94 |

**Table S6.** Summary of ANOVA results for effects of elevated CO_2_ and drought on amino acid contents.

| Measurement | Treatment | *df* | *F* | *P* |
| --- | --- | --- | --- | --- |
|  | CO_2_ | 1 | 3.98 | 0.08 |
| Leucine | Drought | 1 | 0.43 | 0.53 |
|  | CO_2_*drought | 1 | 0.14 | 0.72 |
|  | CO_2_ | 1 | 0.01 | 0.98 |
| Phenylalanine | Drought | 1 | 12.09 | <0.01 |
|  | CO_2_*drought | 1 | 0.53 | 0.49 |
|  | CO_2_ | 1 | 0.28 | 0.61 |
| Alanine | Drought | 1 | 2.72 | 0.14 |
|  | CO_2_*drought | 1 | 28.42 | <0.01 |
|  | CO_2_ | 1 | 8.11 | 0.02 |
| Methionine | Drought | 1 | 3.93 | 0.08 |
|  | CO_2_*drought | 1 | 0.37 | 0.56 |
|  | CO_2_ | 1 | 5.17 | 0.05 |
| Glycine | Drought | 1 | 1.21 | 0.3 |
|  | CO_2_*drought | 1 | 0.06 | 0.81 |
|  | CO_2_ | 1 | 1.18 | 0.31 |
| Glutamate | Drought | 1 | 7.72 | 0.02 |
|  | CO_2_*drought | 1 | 0.01 | 0.94 |
|  | CO_2_ | 1 | 0.01 | 0.92 |
| Glutamine | Drought | 1 | 0.25 | 0.63 |
|  | CO_2_*drought | 1 | 0.92 | 0.37 |
|  | CO_2_ | 1 | 0.01 | 0.95 |
| Valine | Drought | 1 | 5.0 | 0.06 |
|  | CO_2_*drought | 1 | 0.29 | 0.6 |
|  | CO_2_ | 1 | 1.28 | 0.29 |
| Arginine | Drought | 1 | 0.64 | 0.45 |
|  | CO_2_*drought | 1 | 4.21 | 0.07 |
|  | CO_2_ | 1 | 5.83 | 0.04 |
| Lysine | Drought | 1 | 0.22 | 0.65 |
|  | CO_2_*drought | 1 | 1.14 | 0.32 |
|  | CO_2_ | 1 | 0.13 | 0.72 |
| Tyrosine | Drought | 1 | 6.93 | 0.03 |
|  | CO_2_*drought | 1 | 0.79 | 0.4 |
|  | CO_2_ | 1 | 0.37 | 0.56 |
| Proline | Drought | 1 | 14.87 | <0.01 |
|  | CO_2_*drought | 1 | 4.33 | 0.07 |
|  | CO_2_ | 1 | 10.4 | 0.01 |
| Tryptophan | Drought | 1 | 99.59 | <0.01 |
|  | CO_2_*drought | 1 | 6.67 | 0.03 |
|  | CO_2_ | 1 | 0.01 | 0.75 |
| Serine | Drought | 1 | 3.17 | 0.11 |
|  | CO_2_*drought | 1 | 0.56 | 0.47 |
|  | CO_2_ | 1 | 34.46 | <0.01 |
| Threonine | Drought | 1 | 2.6 | 0.15 |
|  | CO_2_*drought | 1 | 3.91 | 0.08 |
|  | CO_2_ | 1 | 5.19 | 0.05 |
| Aspartic acid | Drought | 1 | 7.56 | 0.02 |
|  | CO_2_*drought | 1 | 0.8 | 0.4 |
|  | CO_2_ | 1 | 4.24 | 0.07 |
| Asparagine | Drought | 1 | 9.03 | 0.02 |
|  | CO_2_*drought | 1 | 0.21 | 0.66 |
|  | CO_2_ | 1 | 2.09 | 0.19 |
| Isoleucine | Drought | 1 | 1.04 | 0.34 |
|  | CO_2_*drought | 1 | 0.03 | 0.86 |
|  | CO_2_ | 1 | 5.52 | 0.05 |
| His | Drought | 1 | 0.97 | 0.35 |
|  | CO_2_*drought | 1 | 0.74 | 0.41 |
|  | CO_2_ | 1 | 5.7 | 0.04 |
| Total | Drought | 1 | 10.13 | 0.01 |
|  | CO_2_*drought | 1 | 0.03 | 0.87 |

**Table S7.** Summary of ANOVA results for effects of elevated CO_2_, drought, and aphid infestation on phytohormone contents.

| Measurement | Treatment | *df* | *F* | *P* |
| --- | --- | --- | --- | --- |
|  | CO_2_ | 1 | 2.10 | 0.17 |
|  | Drought | 1 | 141.75 | <0.01 |
|  | Infestation | 1 | 18.19 | <0.01 |
| ABA | CO_2_*drought | 1 | 0.17 | 0.68 |
|  | CO_2_*infestation | 1 | 0.07 | 0.79 |
|  | drought*infestation | 1 | 0.03 | 0.87 |
|  | CO_2_*drought*infestation | 1 | 0.91 | 0.35 |
|  | CO_2_ | 1 | 4.82 | 0.04 |
|  | Drought | 1 | 53.99 | <0.01 |
|  | Infestation | 1 | 6.31 | 0.02 |
| JA | CO_2_*drought | 1 | 3.27 | 0.09 |
|  | CO_2_*infestation | 1 | 0.61 | 0.45 |
|  | drought*infestation | 1 | 0.77 | 0.39 |
|  | CO_2_*drought*infestation | 1 | 0.33 | 0.58 |
|  | CO_2_ | 1 | 35.77 | <0.01 |
|  | Drought | 1 | 0.01 | 0.91 |
|  | Infestation | 1 | 6.87 | 0.02 |
| SA | CO_2_*drought | 1 | 4.6 | 0.05 |
|  | CO_2_*infestation | 1 | 0.7 | 0.42 |
|  | drought*infestation | 1 | 0.85 | 0.37 |
|  | CO_2_*drought*infestation | 1 | 0.01 | 0.99 |

**Table S8.** Summary of ANOVA results for effects of elevated CO_2_, drought, and aphid infestation on JA- and SA-related gene expression.

| Measurement | Treatment | *df* | *F* | | *P* | |
| --- | --- | --- | --- | --- | --- | --- |
|  | CO_2_ | 1 | 2.37 | | 0.14 | |
|  | Drought | 1 | 46.06 | | <0.01 | |
|  | Infestation | 1 | 13.17 | | <0.01 | |
| *AOS* | CO_2_*drought | 1 | 2.02 | | 0.17 | |
|  | CO_2_*infestation | 1 | 0.39 | | 0.54 | |
|  | drought*infestation | 1 | 1.31 | | 0.27 | |
|  | CO_2_*drought*infestation | 1 | 3.62 | | 0.08 | |
|  | CO_2_ | 1 | 5.42 | | 0.03 | |
|  | Drought | 1 | 22.81 | | <0.01 | |
| *LOX* | Infestation | 1 | 61.77 | | <0.01 | |
|  | CO_2_*drought | 1 | 1.78 | | 0.20 | |
|  | CO_2_*infestation | 1 | 0.11 | | 0.74 | |
|  | drought*infestation | 1 | 8.84 | | <0.01 | |
|  | CO_2_*drought*infestation | 1 | 0.39 | | 0.54 | |
|  | CO_2_ | 1 | 62.44 | | <0.01 | |
|  | Drought | 1 | 0.66 | | 0.43 | |
| *PR-1* | Infestation | 1 | 5.49 | 0.03 | |  |
|  | CO_2_*drought | 1 | 0.01 | | 0.95 | |
|  | CO_2_*infestation | 1 | 0.01 | | 0.96 | |
|  | drought*infestation | 1 | 0.32 | | 0.58 | |
|  | CO_2_*drought*infestation | 1 | 0.01 | | 0.92 | |
|  | CO_2_ | 1 | 16.1 | | <0.01 | |
|  | Drought | 1 | 2.98 | | 0.10 | |
| *PAL* | Infestation | 1 | 6.83 | | 0.02 | |
|  | CO_2_*drought | 1 | 0.15 | | 0.70 | |
|  | CO**_2_***infestation | 1 | 0.02 | | 0.88 | |
|  | drought*infestation | 1 | 0.24 | | 0.63 | |
|  | CO_2_*drought*infestation | 1 | 0.63 | | 0.44 | |

**Table S9.** Summary of ANOVA results for effects of elevated CO_2_ and drought on aphid life table parameters.

| Measurement | Treatment | *df* | *F* | *P* |
| --- | --- | --- | --- | --- |
|  | CO_2_ | 1 | 5.97 | 0.04 |
| *R*_0_ | Drought | 1 | 6.62 | 0.03 |
|  | CO_2_*drought | 1 | 0.01 | 0.95 |
|  | CO_2_ | 1 | 3.91 | 0.08 |
| *T* | Drought | 1 | 0.64 | 0.45 |
|  | CO_2_*drought | 1 | 0.02 | 0.90 |
|  | CO_2_ | 1 | 10.19 | 0.01 |
| *r*_m_ | Drought | 1 | 3.75 | 0.09 |
|  | CO_2_*drought | 1 | 0.02 | 0.90 |
|  | CO_2_ | 1 | 10.1 | 0.01 |
| *λ* | Drought | 1 | 3.75 | 0.09 |
|  | CO_2_*drought | 1 | 0.01 | 0.92 |

**Table S10.** Amino acid contents (μg g–1) of wheat grown under heat and drought conditions (mean ±SE, n=3)

| Treatment | Leucine | Phenylalanine | Alanine | Methionine | Glycine |
| --- | --- | --- | --- | --- | --- |
| CK | 21.92±1.93 | 11.98±1.34 | 129.64±12.99 | 4.32±0.61 | 11.91±0.98 |
| Drought | 22.50±2.35 | 17.87±1.39 | 91.53±1.60 | 3.68±0.51 | 11.04±1.43 |
| CO_2_ | 25.23±2.35 | 12.87±1.55 | 69.01±2.09 | 3.27±0.37 | 9.84±0.12 |
| CO_2_*Drought | 27.38±1.60 | 16.83±1.31 | 141.22±15.89 | 2.07±0.33 | 8.46±0.58 |
|  | Glutamate | Glutamine | Valine | Arginine | Lysine |
| CK | 178.89±10.33 | 238.31±28.01 | 26.07±1.90 | 247.57±16.26 | 185.15±11.14 |
| Drought | 221.13±19.39 | 265.31±4.17 | 29.63±1.23 | 195.93±16.95 | 193.51±11.01 |
| CO_2_ | 196.13±11.56 | 254.03±17.88 | 25.07±1.72 | 189.91±16.20 | 166.30±20.04 |
| CO_2_ *Drought | 235.95±16.02 | 245.63±15.5 | 30.94±3.12 | 212.64±22.34 | 144.08±11.64 |
|  | Tyrosine | Proline | Tryptophan | Serine | Threonine |
| CK | 20.59±3.01 | 9.37±0.58 | 38.35±1.09 | 228.79±27.82 | 57.31±1.71 |
| Drought | 29.71±2.31 | 11.26±0.93 | 55.05±2.91 | 209.83±12.95 | 56.32±3.04 |
| CO_2_ | 23.84±2.79 | 7.70±1.16 | 26.02±2.48 | 248.53±16.79 | 36.13±2.63 |
| CO_2_ *Drought | 28.35±2.15 | 14.14±1.42 | 53.70±1.31 | 206.98±11.39 | 45.82±3.17 |
|  | Aspartic acid | Asparagine | Isoleucine | Histidine | Total |
| CK | 48.37±4.65 | 1370.98±194.5 | 37.74±2.08 | 191.59±14.65 | 3053.85±197.39 |
| Drought | 54.57±3.18 | 1956.7±183.31 | 35.68±2.58 | 217.44±17.66 | 3678.71±250.83 |
| CO_2_ | 37.80±2.84 | 1098.83±97.28 | 36.73±2.63 | 170.80±14.25 | 2638.39±133.20 |
| CO_2_ *Drought | 49.94±2.19 | 1531.01±183.99 | 38.79±2.50 | 172.52±7.29 | 3022.21±141.13 |
